# We Should Pay More Attention to Cross-sectoral Cooperation, Incentives and Practice-Oriented Evaluation: Comment on "The Effect of Integrated Care After Discharge From Hospitals on Outcomes Among Korean Older Adults"

**DOI:** 10.34172/ijhpm.9088

**Published:** 2025-10-01

**Authors:** Eva Blozik

**Affiliations:** ^1^Institute of Primary Care, University of Zürich, Zürich, Switzerland.; ^2^Institute of Primary Care, University Hospital of Zürich, Zürich, Switzerland.

**Keywords:** Integrated Care, Evaluation, Implementation, Quality of Care, Incentives, Collaboration

## Abstract

In their study, Choi and Yoo analysed a pilot project of the Koran government providing integrated care of older adults discharged from hospitals. The pilot project resulted in reduced length of home stay, cost reduction in total expenses and emergency visits for participants compared to controls, but increased hospital readmissions. This commentary provides actionable insights for stakeholders and researchers interested in testing, implementing and monitoring integrated care services in routine care settings. An essential requirement is that barriers and success factors of such projects need to be addressed in scientific evaluations. Also, understanding implementation projects as a continuous quality improvement process may increase knowledge gain and practical relevance of innovative initiatives. Furthermore, heterogeneity across healthcare providers, patient populations, and regions should be considered an asset from which different solutions can emerge. Regulatory authorities are in the position to create a framework so that stakeholders have the required scope for action.

## Introduction

 In their study, Choi and Yoo analysed the outcomes of a pilot project of the Korean government providing integrated care of older adults discharged from hospitals.^1^ Integrated care services included housekeeping support, nutrition support, movement assistance, home repair, consultations and education for healthy lifestyle, and some home-based primary care depending on regional availability of healthcare service providers.

 The analyses included a total of 1895 older adults who participated in the pilot project between August 1, 2019 and April 30, 2022 in comparison to a clinically somewhat different group of 7145 older adults who lived in regions where no pilot project was available using propensity score matching.

 The length of home stay, total expenses of national health insurance and long-term care insurance, emergency visits, and hospital readmission for the same disease were measured and compared using difference-in-differences analysis.

 The analyses revealed favourable effects of integrated care for length of home stay, cost reduction in total healthcare and long-term care expenses and emergency visits for participants of the pilot project compared to controls. However, participants were significantly more frequently rehospitalized for the same disease. The authors concluded that the integrated care after discharge from hospitals can help older adults to continue living in the place where they lived, and improved collaboration between clinics and hospitals is required to prevent readmissions.

## Purpose of Integrated Care

 Integrated care addresses the undesirable effects of healthcare systems that have historically grown in a fragmented way and are divided by different care sectors and reimbursement incentives. The aim of integrated care is to increase the quality and efficiency of healthcare by focusing on patient-sided outcomes on the patient’s treatment path, facilitating information transfer, communication and coordination between the various healthcare providers involved, and by addressing prevention and health promotion as well as non-medical factors related to maintaining health, recovering from illness, and living with limitations.^2^

## Intersectoral and Interprofessional Cooperation and Collaboration

 The paper published by Choi and Yoo apparently focused on supporting geriatric patients to stay at home as independent as possible, on preserving older person’s own resources and abilities and on providing a basic level of primary care. The results of the pilot project support the causal hypothesis that integrated care is effective in improving home stay, and – as a result of increased home stay, total cost. However, certain essential aspects of integrated care were not mentioned in the manuscript. Specifically, it is unclear whether there were agreements regarding care processes between the inpatient providers and the subsequent outpatient providers, whether a smooth transfer of information between healthcare settings was ensured, whether the inpatient and outpatient care sectors followed agreed evidence-based guidelines, and whether there was an explicit willingness to cooperate among the care providers involved in the care of project participants. If those aspects were neglected, it is also plausible that no benefit was generated in the project for hospital readmission. The diverse services may even have led to insufficient attention being paid to the need for medical intervention. It may no longer have been clear who was the central contact person in lead for providing medical care. The results of Choi and Yoo indicate the need to clarify cross-sectoral cooperation and interfaces in order to achieve sustainable improvements in quality of healthcare.^3^

 Multiple experience from implementation projects shows that implementing integrated care requires not only a solid evidence-based foundation, but also, to a certain extent, a change of mindset.^4^ Collaboration across sectoral boundaries requires trust and recognition of each other’s competencies, no fear of losing skills or earning opportunities, a departure from traditional role understandings, and a focus on the patient’s outcome, which may be achieved long after the patient is no longer being treated in their own setting.^5^ However, for the implementation of integrated care models, it is recommended that in addition to defining specific changes in the care process, a focus needs to be placed on the necessary accompanying change management.^6^

## Incentives and Governance

 Another relevant question is whether incentives are designed to spur the implementation of integrated care. In the present pilot project by Choi and Yoo, it remains unclear how remuneration was structured in the project and whether the monetary interests of the stakeholders involved in the care of project participants were aligned. Traditional reimbursement systems reward quantity over quality. If the quality of outpatient care is increased, leading to fewer unjustified events such as readmissions, inpatient case numbers and revenues will decline.

 In order to advance integrated care models, innovative forms of financing are needed, with which an increase in quality or efficiency on the patient pathway also benefits all who have contributed to this result. This is where shared benefit models come in.^7,8^ Future pilot projects should explicitly address the aspect of remuneration; and this aspect should also be explicitly reported in relevant scientific evaluations.

 Monitoring quality indicators, outcomes and costs is not only necessary as a basis for shared benefit models but is also an essential requirement for quality management processes.^9^ Quality can only be improved if stakeholders know the starting point and can see the impact of their actions on quality and efficiency. Implementation of integrated care models therefore should be accompanied by monitoring and evaluation efforts.^10^ For example, a chronic care management program in Swiss primary care implemented in the context of managed care insurance models including evidence-based and interdisciplinary procedures, incentives and a log-term evaluation and quality improvement process showed to be highly effective in increasing quality of care, patient and provider satisfaction with care processes and in reducing healthcare cost.^11^ Previous research in Swiss primary care indicated that incentives to ameliorate in certain primary care quality indicators leads to improvement in the incentivized parameters. However, the effect of the (relatively small) incentives seemed to be less pronounced than the effect of the accompanying data transparency.^12,13^

## Testing Integrated Care Approaches in Real-Life Settings

 It should also be noted that implementation projects aimed at increasing the quality or efficiency of care take place under real-life conditions. They aim to introduce new methods, new processes, new ways of thinking into care routines. It seems obvious that such attempts cannot be done perfectly in the first attempt but require several cycles of improvement before the desired results are achieved.

 A before-after comparison that does not examine the potential factors for project success, the obstacles and success factors does not do justice to the claim of iterative improvement of such complex interventions. Thus, it is unlikely that the pilot tested by Choi and Yoo already represents the achievable maximum of the integrated care approach, but rather its first implementation attempt. An established research method to evaluate “what works, for whom, under what circumstances and how” is called realist evaluation.^14^ The aim of this method is to consider context information such as economic, geographic, historical, social, and political circumstances as determining factors for successful implementation mechanisms for achieving positive project outcomes. Realist evaluation is aimed at increasing transparency about the complex processes underlying implementation initiatives in healthcare. It can indicate situations in which the intervention of interest works (or not). This should allow decision-makers to assess whether interventions tested in one setting may be successful in another setting and it supports programme planners in adapting interventions to fit to their specific contexts. If the principles of realistic evaluation were more systematically taken into account in the evaluation of integrated care implementation projects, the resulting research results would be much more easily put into practice.

## Conclusion

 In summary, Choi and Yoo provide an example from the field of geriatric care showing that integrated care significantly reduces the cost of care offering significant potential for a triple win situation for patients, providers and payers. However, integrated care models should explicitly address aspects of collaboration in order to be successful and safe for patients.

 In order to maximize the knowledge gained from pilot experiments, researchers should systematically investigate and share the obstacles and success factors of their project, view the project as a continuous process of further development and pay sufficient attention to incentive factors.

## Ethical issues

 Not applicable.

## Conflicts of interest

 EV is affiliated with SWICA Healthcare Organization, a health insurance engaged in fostering integrated care and providing managed care models, including integrated care elements. She also engaged in VBHC Suisse (vbhc.ch), a nonprofit association that aims to foster the value-based transformation of the Swiss healthcare system.
